# Supplementary material for: Iterative Multidisciplinary Development and Evaluation of a Patient-Facing Social Determinants of Health Chatbot Using Synthetic Data Simulation: Mixed Methods Study
Source: JMIR Form Res. 2026 Aug 7;10:e89837. doi: 10.2196/89837 (PMC13450882; doi:10.2196/89837)
Supplement: Supplementary file 1 [file formative-v10-e89837-s004.pdf]

Reporting Checklists

GRAMMS: Good Reporting of A Mixed Methods Study[1]

Reporting item	Reported	Location in manuscript (section and opening phrase)
1. Describe the justification for using a mixed methods approach to the research question	**Yes**	Methods, Study Design — “A convergent mixed-methods design was selected because…”
2. Describe the design in terms of the purpose, priority and sequence of methods	**Yes**	Methods, Study Design — “We conducted a formative mixed-methods feasibility study using…”
3. Describe each method in terms of sampling, data collection and analysis	**Yes**	Methods, Study Design ("We conducted..."); details in Clinical Scenario Development, Simulation Protocol...
4. Describe where integration has occurred, how it has occurred and who has participated in it	**Yes**	Analysis, Mixed Methods Analysis — “Quantitative and qualitative findings were integrated at the interpretation stage…”
5.Describe any limitation of one method associated with the presence of the other method	**Yes**	Strengths and Limitations — “Integration and interpretation of findings were performed by team members who also developed…”
6. Any insights gained from mixing or integrating methods	**Yes**	Results, Rater Agreement Across Domains — “Integration of the two data sources produced insights that neither yielded alone…”

SRQR: Standards for Reporting Qualitative Research[2]

Reporting item	Reported	Location in manuscript (section and opening phrase)
1. Title	**Yes**	Title page
2. Abstract	**Yes**	Abstract
3. Problem formulation	**Yes**	Introduction: “Social determinants of health (SDoH) account for 80%…”
4. Purpose or research question	**Yes**	Introduction (Objective): “Our objective is to describe a highly feasible…”
5. Qualitative approach and research paradigm	**Yes**	Analysis, Qualitative Analysis: “We employed a rapid qualitative analysis approach…”
6. Researcher characteristics and reflexivity	**Yes**	Methods / Strengths and Limitations: “All raters were internal members of the research team who also contributed to development…”
7. Context	**Yes**	Clinical Scenario Development: “Twenty-seven synthetic clinical scenarios were developed by an emergency medicine physician…”
8. Sampling strategy	**Yes**	Clinical Scenario Development (“Twenty-seven synthetic clinical scenarios were developed…”); raters at Multidisciplinary Evaluation Process (“Three internal evaluators from the research team…”)
9. Ethical issues pertaining to human subjects	**Yes**	Ethics Approval and Consent to Participate: “This study used synthetic clinical scenarios and role-play simulation…”
10. Data collection methods	**Yes**	Simulation Protocol: “The final prompt designed in the Chatbot Development and Refinement phase was provided…”
11. Data collection instruments and technologies	**Yes**	Rubric Development; Appendices: “We developed a 10-criterion evaluation rubric, called Patient-Facing Healthcare Chatbot…”
12. Units of study	**Yes**	Multidisciplinary Evaluation Process; Results: “Three internal evaluators from the research team…”
13. Data processing	**Yes**	Analysis, Qualitative Analysis: “Qualitative comments were organized into a matrix across rubric domains and raters…”
14. Data analysis	**Yes**	Analysis, Qualitative Analysis: “We employed a rapid qualitative analysis approach…”
15. Techniques to enhance trustworthiness	**Yes**	Analysis, Qualitative Analysis: “Trustworthiness was supported through investigator triangulation across the three clinical disciplines…”
16. Synthesis and interpretation	**Yes**	Results, Rater Agreement Across Domains: “Analysis of both quantitative rubric scores and qualitative feedback revealed agreement…”
17. Links to empirical data (illustrative quotes)	**Yes**	Results, Rater Agreement Across Domains: “These differing expectations were reflected in rater comments…”
18. Integration with prior work, implications, transferability, contribution	**Yes**	Discussion: “While recent frameworks, such as the Health Care AI Chatbot Evaluation Framework…”

Reporting item	Reported	Location in manuscript (section and opening phrase)
19. Limitations	**Yes**	Strengths and Limitations: “Our approach demonstrates both important strengths and limitations…”
20. Conflicts of interest	**Yes**	Conflicts of Interest: “AL is a co-founder of an entity that intends to deploy the chatbot…”
21. Funding	**Yes**	Funding statement

DECIDE-AI — Early-Stage Clinical Evaluation of AI Decision-Support Systems[3]

Reporting item	Reported	Location in manuscript (section and opening phrase)
1. Title (AI-specific)	**Yes**	Title page
2. Intended use	**Yes**	Intended Clinical Role of the Chatbot: “The chatbot was designed as a screening support tool, not an autonomous…”
3. Participants — patients and users (recruitment, criteria, training)	**Not applicable**	*Synthetic scenarios; no real patients or end-users were enrolled. One licensed clinician role-played all cases. Real participants are deferred to future phases.*
4. AI system (description/version/algorithm; inputs; outputs)	**Yes**	Chatbot Development and Refinement (“We iteratively refined a prompt using common prompt engineering techniques…”); inputs/outputs at “Within this workflow, the system inputs were simulated-patient utterances…”
5. Implementation (settings; workflow; timing; who decides)	**Yes**	Intended Clinical Role of the Chatbot (“The chatbot was designed as a screening support tool…”); Strengths and Limitations (“Consistent with DECIDE-AI, several implementation factors remain unevaluated…”)
6. Safety and errors — definition and identification	**Yes**	Analysis, Quantitative Analysis: “For the safety domain, a criterion was scored as not met when an evaluator judged…”
7. Human factors (tools, methods, frameworks, users)	**Not applicable**	*No usability or human-factors evaluation in this pre-deployment phase; planned as future work.*
8. Ethics (methodologies for an ethics goal, e.g., fairness)	**Yes**	Clinical Scenario Development (equity ‘stress-test’ scenarios) : “expanded the evaluation beyond these CMS domains…”
I. Abstract	**Yes**	Abstract
II. Objectives	**Yes**	Introduction (Objective) : “Our objective is to describe a highly feasible…”
III. Research governance (protocol, registration, ethics)	**Yes**	Ethics Approval and Consent to Participate (COMIRB #22-1944) : “This project was part of a larger study which was approved by the…”
IV. Outcomes	**Yes**	Methods, Study Design : “Primary outcomes were (a) proportion of rubric criteria met…”
V. Analysis	**Yes**	Analysis, Quantitative Analysis : “To characterize interrater reliability for binary rubric items, we report…”
VI. Patient involvement	**Yes**	Patient and Public Involvement : “Patients were not involved in the design or conduct of this phase…”
9. Participants — baseline characteristics (results)	**Not applicable**	*No real participants; synthetic scenario characteristics are reported in the Results instead.*
10. Implementation — exposure, adherence, workflow change (results)	**Not applicable**	*Not measurable in a simulated, pre-deployment evaluation.*

Reporting item	Reported	Location in manuscript (section and opening phrase)
11. Modifications to the AI system during the study	**Yes**	Chatbot Development and Refinement: No modifications were made during the reported evaluation. Prompt refinement occurred before the final evaluation phase and is described in the Chatbot Development and Refinement section
12. Human–computer agreement	**Not applicable**	*No clinician users acted on AI recommendations; rater agreement is a distinct construct and is reported separately.*
13. Safety and errors (results)	**Yes**	Results: "Safety performance was more variable overall…"
14. Human factors — usability, learning curves (results)	**Not applicable**	*Not evaluated in this phase; planned as future work.*
VII. Main results	**Yes**	Results: "The chatbot received high proportions of positive ratings across several domains…"
VIII. Subgroup analysis	**Yes**	Results: "with lower scores concentrated in urgent social needs scenarios…"
15. Support for intended use (discussion)	**Yes**	Discussion: "This distinction has implications for deployment…"
16. Safety and errors (discussion)	**Yes**	Discussion (safety-domain reframing): "Prompted by review of the qualitative and quantitative data…"
IX. Strengths and limitations	**Yes**	Strengths and Limitations: "Our approach demonstrates both important strengths and limitations…"
17. Data availability	**Yes**	Data Availability; Appendices: "The synthetic scenarios, the PF-HCER rubric with rater instructions, and the full system prompt are provided…"
X. Conflicts of interest	**Yes**	Conflicts of Interest: "AL is a co-founder of an entity that intends to deploy the chatbot…"

[1] O'Cathain A, Murphy E, Nicholl J. The quality of mixed methods studies in health services research. J Health Serv Res Policy 2008;13:92–8.

[2] O'Brien BC, Harris IB, Beckman TJ, Reed DA, Cook DA. Standards for reporting qualitative research: a synthesis of recommendations. Acad Med 2014;89:1245–51.

[3] Vasey B, Nagendran M, Campbell B, Clifton DA, Collins GS, Denaxas S, et al. Reporting guideline for the early stage clinical evaluation of decision support systems driven by artificial intelligence: DECIDE-AI. BMJ 2022;377:e070904.
