# Supplementary material for: Iterative Multidisciplinary Development and Evaluation of a Patient-Facing Social Determinants of Health Chatbot Using Synthetic Data Simulation: Mixed Methods Study
Source: JMIR Form Res. 2026 Aug 7;10:e89837. doi: 10.2196/89837 (PMC13450882; doi:10.2196/89837)
Supplement: Supplementary file 4 [file formative-v10-e89837-s003.docx]

**Appendix 3.**

| **Table 1. Chatbot Performance Across Scenario Types** | | | | | | | | | | |
| --- | --- | --- | --- | --- | --- | --- | --- | --- | --- | --- |
| **Case Characteristic** | **Accuracy** | | | | **Trustworthiness** | | | | **Empathy** | |
|  | **Domain Focus &** **Completeness** | **Accurate** **Interpretation** | **Communication** **Quality &** **Cultural** **Sensitivity** | **Appropriately** **Adaptive** | **Safety** | **Privacy** | **Bias** | **Role** **Adherence** | **Completeness** **of Data** **Capture** | **Patient** **Engagement** **& Experience** |
|  | % Agreement (95% CI) Fleiss' κ (95% CI) | % Agreement (95% CI) Fleiss' κ (95% CI) | % Agreement (95% CI) Fleiss' κ (95% CI) | % Agreement (95% CI) Fleiss' κ (95% CI) | % Agreement (95% CI) Fleiss' κ (95% CI) | % Agreement (95% CI) Fleiss' κ (95% CI) | % Agreement (95% CI) Fleiss' κ (95% CI) | % Agreement (95% CI) Fleiss' κ (95% CI) | % Agreement (95% CI) Fleiss' κ (95% CI) | % Agreement (95% CI) Fleiss' κ (95% CI) |
| **Overall Performance (n=27)** | 0.51 (0.32–0.69) 0.21 (-0.06–0.48) | 0.98 (0.92–1.03) -0.03 (-0.29–0.24) | 0.99 (0.95–1.03) -0.01 (-0.28–0.25) | 0.99 (0.95–1.03) -0.01 (-0.28–0.25) | 0.69 (0.52–0.87) -0.04 (-0.31–0.23) | 0.95 (0.87–1.03) -0.05 (-0.32–0.21) | 1.00 (1.00–1.00) N/A | 0.99 (0.95–1.03) -0.01 (-0.28–0.25) | 0.59 (0.41–0.78) 0.18 (-0.08–0.45) | 0.93 (0.83–1.02) -0.08 (-0.35–0.19) |
| **SDoH Domain** | | | | | | | | | | |
| Financial (n=4) | 0.50 (0.01–0.99) 0.33 (-0.36–1.03) | 0.92 (0.65–1.19) -0.09 (-0.78–0.60) | 1.00 (1.00–1.00) N/A | 1.00 (1.00–1.00) N/A | 0.92 (0.65–1.19) -0.09 (-0.78–0.60) | 1.00 (1.00–1.00) N/A | 1.00 (1.00–1.00) N/A | 1.00 (1.00–1.00) N/A | 0.75 (0.33–1.17) 0.11 (-0.58–0.80) | 0.92 (0.65–1.19) -0.09 (-0.78–0.60) |
| Transportation (n=4) | 0.67 (0.20–1.13) 0.63 (-0.07–1.32) | 1.00 (1.00–1.00) N/A | 1.00 (1.00–1.00) N/A | 1.00 (1.00–1.00) N/A | 0.83 (0.47–1.20) -0.20 (-0.89–0.49) | 1.00 (1.00–1.00) N/A | 1.00 (1.00–1.00) N/A | 1.00 (1.00–1.00) N/A | 0.83 (0.47–1.20) -0.20 (-0.89–0.49) | 0.92 (0.65–1.19) -0.09 (-0.78–0.60) |
| Mental Health (n=2) | 0.50 (-0.19–1.19) -0.33 (-1.31–0.65) | 1.00 (1.00–1.00) N/A | 1.00 (1.00–1.00) N/A | 0.83 (0.32–1.35) -0.20 (-1.18–0.78) | 0.67 (0.01–1.32) -0.50 (-1.48–0.48) | 0.83 (0.32–1.35) -0.20 (-1.18–0.78) | 1.00 (1.00–1.00) N/A | 1.00 (1.00–1.00) N/A | 0.33 (-0.32–0.99) -0.50 (-1.48–0.48) | 0.67 (0.01–1.32) -0.50 (-1.48–0.48) |
| Housing (n=3) | 0.56 (-0.01–1.12) -0.35 (-1.15–0.45) | 0.89 (0.53–1.24) -0.12 (-0.93–0.68) | 0.89 (0.53–1.24) -0.12 (-0.93–0.68) | 1.00 (1.00–1.00) N/A | 0.78 (0.31–1.25) -0.29 (-1.09–0.51) | 1.00 (1.00–1.00) N/A | 1.00 (1.00–1.00) N/A | 1.00 (1.00–1.00) N/A | 0.67 (0.13–1.20) -0.00 (-0.80–0.80) | 1.00 (1.00–1.00) N/A |
| Interpersonal Safety (n=3) | 0.44 (-0.12–1.01) -0.35 (-1.15–0.45) | 1.00 (1.00–1.00) N/A | 1.00 (1.00–1.00) N/A | 1.00 (1.00–1.00) N/A | 0.33 (-0.20–0.87) -0.50 (-1.30–0.30) | 0.78 (0.31–1.25) -0.29 (-1.09–0.51) | 1.00 (1.00–1.00) N/A | 1.00 (1.00–1.00) N/A | 0.67 (0.13–1.20) -0.00 (-0.80–0.80) | 0.89 (0.53–1.24) -0.13 (-0.93–0.68) |
| Substance Use (n=5) | 0.20 (-0.15–0.55) 0.17 (-0.45–0.79) | 1.00 (1.00–1.00) N/A | 1.00 (1.00–1.00) N/A | 1.00 (1.00–1.00) N/A | 0.73 (0.35–1.12) -0.02 (-0.64–0.60) | 1.00 (1.00–1.00) N/A | 1.00 (1.00–1.00) N/A | 0.93 (0.71–1.15) -0.07 (-0.69–0.55) | 0.20 (-0.15–0.55) -0.25 (-0.87–0.37) | 0.93 (0.71–1.15) -0.07 (-0.69–0.55) |
| Language Barrier/Health Literacy (n=2) | 0.83 (0.32–1.35) -0.20 (-1.18–0.78) | 1.00 (1.00–1.00) N/A | 1.00 (1.00–1.00) N/A | 1.00 (1.00–1.00) N/A | 0.83 (0.32–1.35) -0.20 (-1.18–0.78) | 0.83 (0.32–1.35) -0.20 (-1.18–0.78) | 1.00 (1.00–1.00) N/A | 1.00 (1.00–1.00) N/A | 1.00 (1.00–1.00) N/A | 1.00 (1.00–1.00) N/A |
| Multiple SDOH (n=4) | 0.58 (0.10–1.07) 0.31 (-0.38–1.01) | 1.00 (1.00–1.00) N/A | 1.00 (1.00–1.00) N/A | 1.00 (1.00–1.00) N/A | 0.42 (-0.07–0.90) -0.37 (-1.06–0.32) | 1.00 (1.00–1.00) N/A | 1.00 (1.00–1.00) N/A | 1.00 (1.00–1.00) N/A | 0.50 (0.01–0.99) -0.00 (-0.69–0.69) | 1.00 (1.00–1.00) N/A |
| **Age** | | | | | | | | | | |
| Teen (<19) (n=7) | 0.43 (0.06–0.80) -0.17 (-0.69–0.36) | 1.00 (1.00–1.00) N/A | 1.00 (1.00–1.00) N/A | 1.00 (1.00–1.00) N/A | 0.52 (0.15–0.89) -0.15 (-0.67–0.38) | 0.90 (0.69–1.12) -0.11 (-0.63–0.42) | 1.00 (1.00–1.00) N/A | 1.00 (1.00–1.00) N/A | 0.52 (0.15–0.89) -0.15 (-0.67–0.38) | 0.95 (0.79–1.11) -0.05 (-0.57–0.47) |
| Young Adult (19–39) (n=7) | 0.59 (0.27–0.91) 0.39 (-0.08–0.85) | 0.93 (0.75–1.10) -0.08 (-0.54–0.38) | 0.96 (0.84–1.09) -0.04 (-0.50–0.42) | 1.00 (1.00–1.00) N/A | 0.85 (0.62–1.08) 0.12 (-0.34–0.58) | 1.00 (1.00–1.00) N/A | 1.00 (1.00–1.00) N/A | 0.96 (0.84–1.09) -0.04 (-0.50–0.42) | 0.67 (0.36–0.97) 0.33 (-0.13–0.80) | 0.89 (0.68–1.09) -0.12 (-0.59–0.34) |
| Middle Age  (40–64)  (n=3) | 0.44 (-0.12–1.01) 0.10 (-0.70–0.90) | 1.00 (1.00–1.00) N/A | 1.00 (1.00–1.00) N/A | 1.00 (1.00–1.00) N/A | 0.44 (-0.12–1.01) -0.35 (-1.15–0.45) | 1.00 (1.00–1.00) N/A | 1.00 (1.00–1.00) N/A | 1.00 (1.00–1.00) N/A | 0.22 (-0.25–0.69) -0.29 (-1.09–0.51) | 1.00 (1.00–1.00) N/A |
| Older Adult  (65+)  (n=8) | 0.50 (0.15–0.85) 0.33 (-0.16–0.82) | 1.00 (1.00–1.00) N/A | 1.00 (1.00–1.00) N/A | 0.96 (0.82–1.10) -0.04 (-0.53–0.45) | 0.75 (0.45–1.05) -0.33 (-0.82–0.16) | 0.92 (0.73–1.11) -0.09 (-0.58–0.40) | 1.00 (1.00–1.00) N/A | 1.00 (1.00–1.00) N/A | 0.71 (0.39–1.02) 0.19 (-0.30–0.68) | 0.92 (0.73–1.11) -0.09 (-0.58–0.40) |
| **Gender** | | | | | | | | | | |
| Male (n=13) | 0.49 (0.22–0.76) 0.18 (-0.21–0.56) | 1.00 (1.00–1.00) N/A | 1.00 (1.00–1.00) N/A | 1.00 (1.00–1.00) N/A | 0.67 (0.41–0.92) -0.04 (-0.42–0.35) | 0.95 (0.83–1.07) -0.05 (-0.44–0.33) | 1.00 (1.00–1.00) N/A | 0.97 (0.89–1.06) -0.03 (-0.41–0.36) | 0.59 (0.32–0.86) 0.26 (-0.13–0.64) | 0.97 (0.89–1.06) -0.03 (-0.41–0.36) |
| Female (n=13) | 0.51 (0.24–0.78) 0.28 (-0.10–0.67) | 0.97 (0.89–1.06) -0.03 (-0.41–0.36) | 1.00 (1.00–1.00) N/A | 0.97 (0.89–1.06) -0.03 (-0.41–0.36) | 0.69 (0.44–0.94) -0.08 (-0.47–0.30) | 0.95 (0.83–1.07) -0.05 (-0.44–0.33) | 1.00 (1.00–1.00) N/A | 1.00 (1.00–1.00) N/A | 0.56 (0.29–0.83) 0.06 (-0.32–0.45) | 0.87 (0.69–1.05) -0.15 (-0.53–0.24) |
| Nonbinary (n=1) | 0.67 -0.50 (-1.89–0.89) | 0.67 -0.50 (-1.89–0.89) | 0.67 -0.50 (-1.89–0.89) | 1.00 N/A | 1.00 N/A | 1.00 N/A | 1.00 N/A | 1.00 N/A | 1.00 N/A | 1.00 N/A |
| **Urgent Social Needs (n=4)** | 0.33 (-0.13–0.80) -0.13 (-0.82–0.57) | 1.00 (1.00–1.00) N/A | 1.00 (1.00–1.00) N/A | 0.92 (0.65–1.19) -0.09 (-0.78–0.60) | 0.42 (-0.07–0.90) -0.37 (-1.06–0.32) | 0.83 (0.47–1.20) -0.20 (-0.89–0.49) | 1.00 (1.00–1.00) N/A | 1.00 (1.00–1.00) N/A | 0.58 (0.10–1.07) -0.03 (-0.72–0.66) | 0.83 (0.47–1.20) -0.20 (-0.89–0.49) |
| *Note: Each cell shows % Agreement (mean proportion of Yes ratings) above and Fleiss’ κ below, both with analytic 95% confidence intervals. % Agreement CI: PA ± 1.96×SE(PA), where SE(PA)=√(PA×(1−PA)/N). Kappa CI: κ ± 1.96×SE(κ), where SE(κ)=√(2×(p_j×(1−p_j))²/(N×(1−P_e)²)) (Fleiss, Cohen & Everitt, 1969). N/A = insufficient variance to compute κ (unanimous agreement) or SE (N=1).* | | | | | | | | | | |
